# Supplementary material for: Structural basis of the activation of type 1 insulin-like growth factor receptor
Source: Nat Commun. 2019 Oct 8;10:4567. doi: 10.1038/s41467-019-12564-0 (PMC6783537; doi:10.1038/s41467-019-12564-0)
Supplement: Supplementary file 1 — Supplementary Information [file 41467_2019_12564_MOESM1_ESM.pdf]

Supplementary Information

Structural basis of the activation of type 1 insulin-like growth factor receptor

Li *et al.*

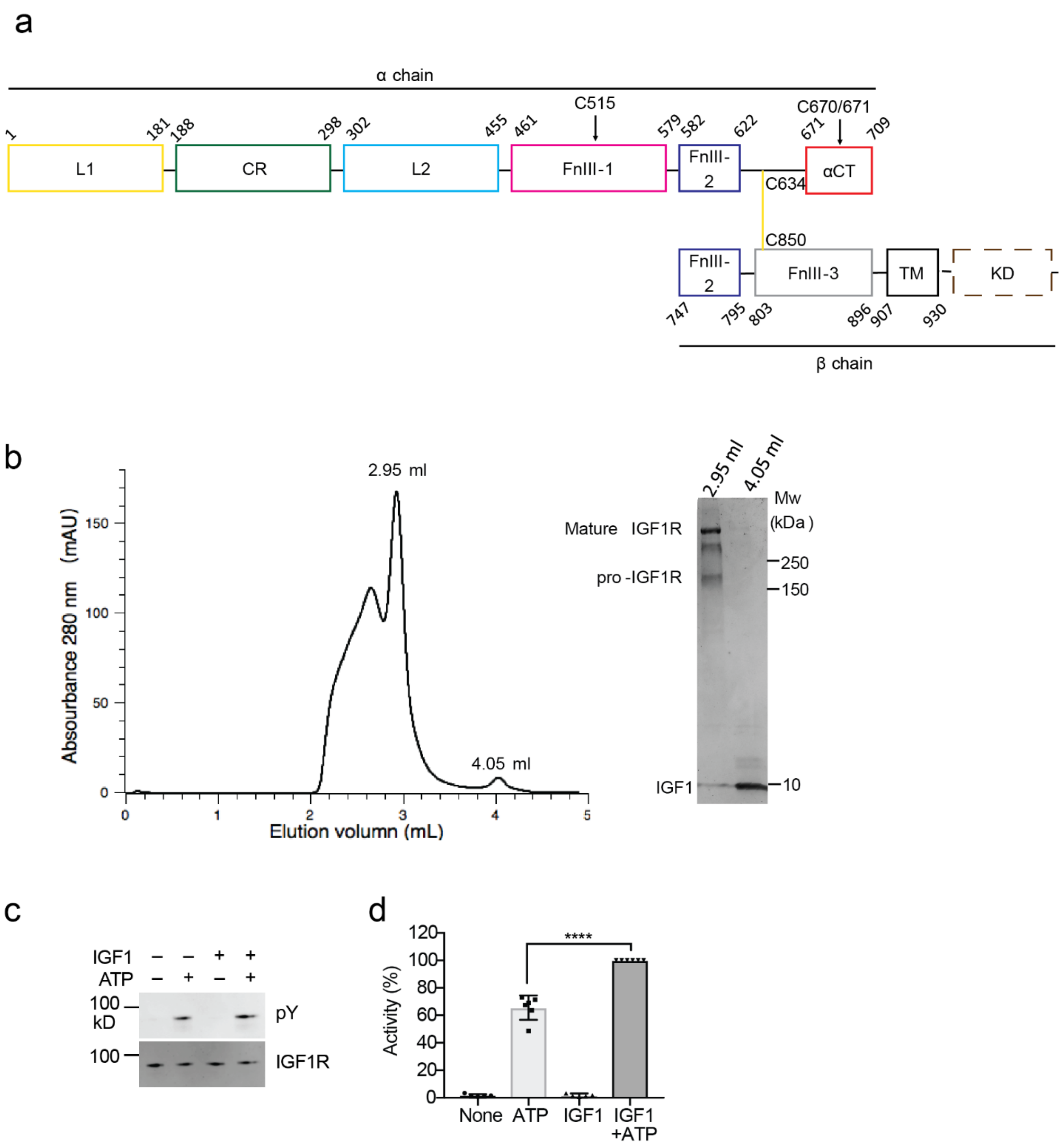

Supplementary Fig. 1 | Purification of the full-length MmIGF1R. a, Domain organization of IGF1R. **b**, Representative size-exclusion chromatogram of IR. The peak fractions were visualized on SDS–PAGE by Coomassie staining. **c**, *In vitro* phosphorylation assay using the recombinant mouse IGF1R isolated with detergent. **d**, Quantification of the *in vitro* phosphorylation assay shown in **c** (Mean ± SD). Each experiment was repeated six times. Significance calculated using two-tailed students *t*-test; ****p<0.0001. Source data are provided as a Source Data file.

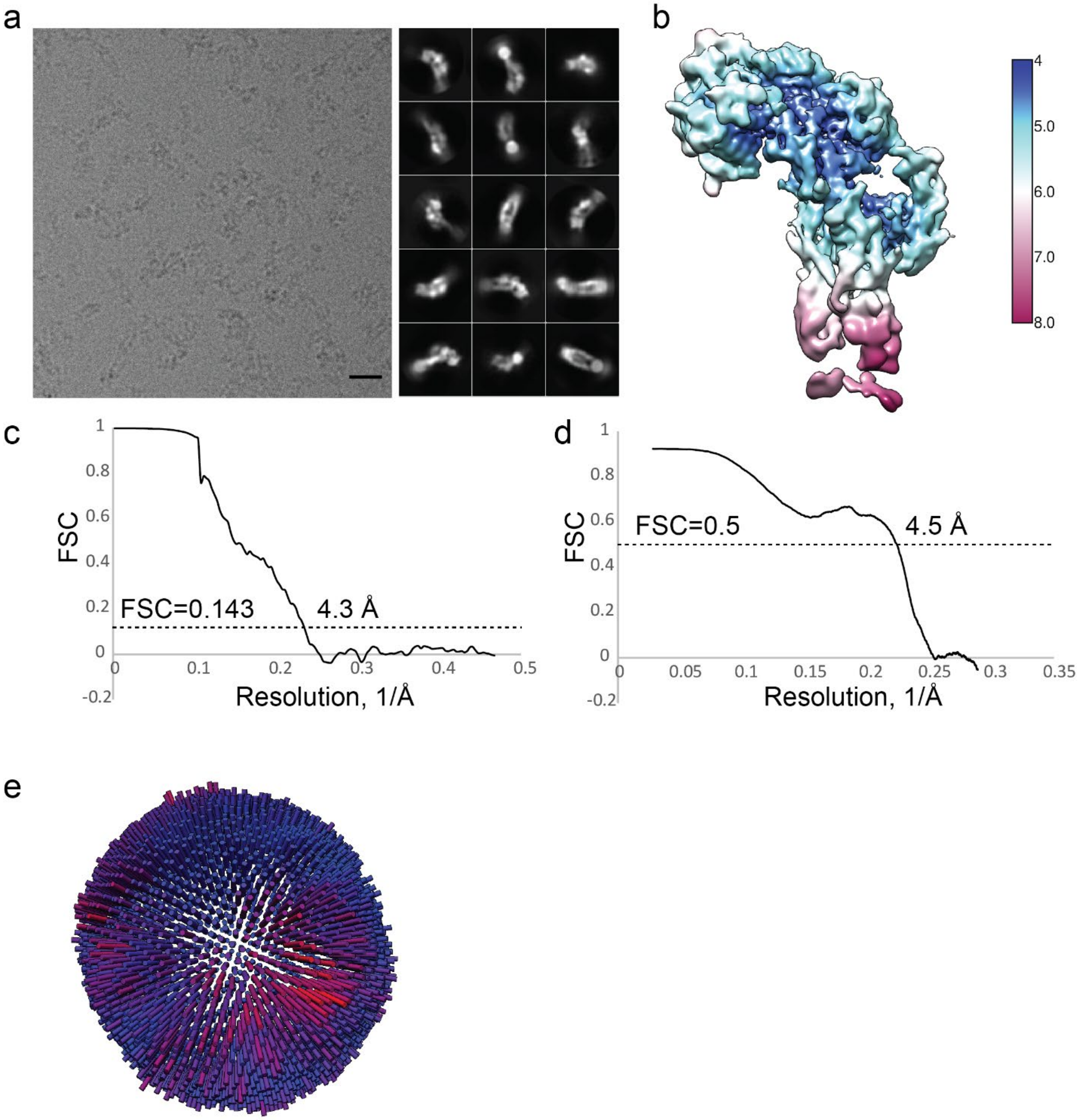

Supplementary Fig. 2 | Cryo-EM analysis of the IGF1R-IGF1 complex. a, Representative electron micrograph and 2D class averages of the IGF1R-IGF1 complex. **b**, unsharpened cryo-EM map colored by local resolution. **c**, The gold-standard Fourier shell correlation curve for the cryo-EM map shown in Fig. 1a. **d**, FSC curves for the refined model versus the map. **e**, Euler angle distribution of particles used in the final 3D reconstructions. Scale bar: 25 nm.

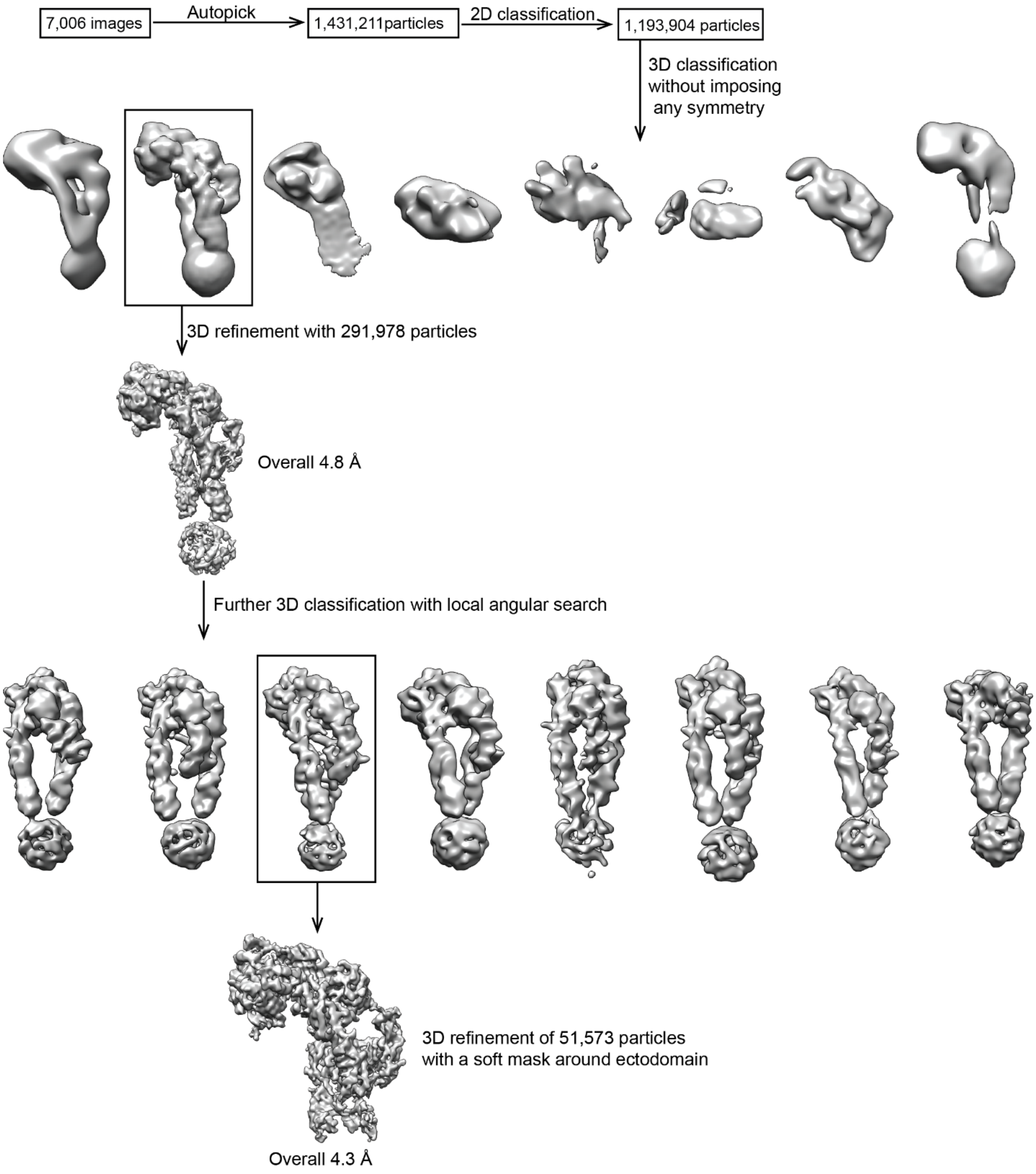

Supplementary Fig. 3 | Flowchart of cryo-EM data processing.

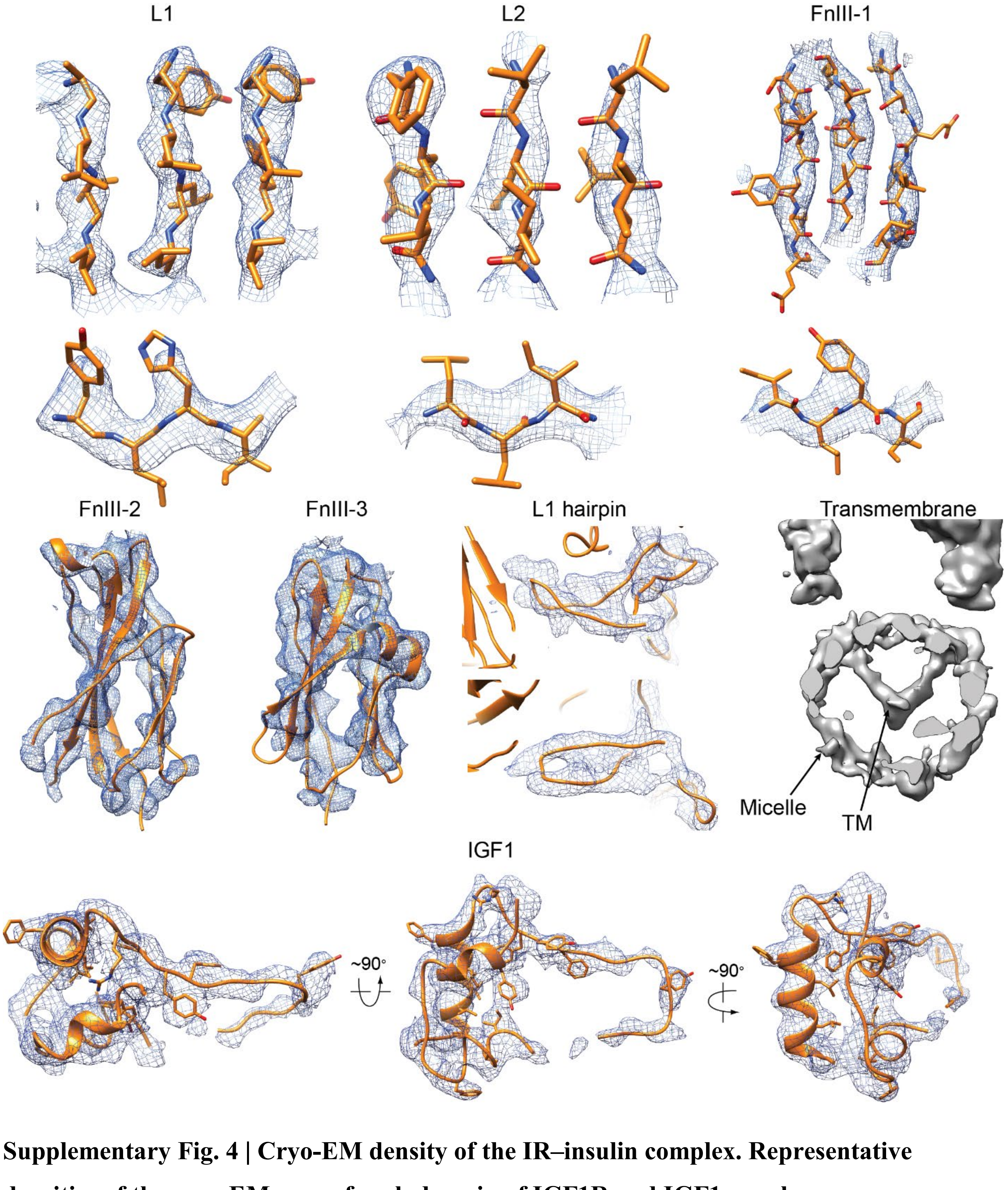

Supplementary Fig. 4 | Cryo-EM density of the IR–insulin complex. Representative densities of the cryo-EM map of each domain of IGF1R and IGF1 are shown.

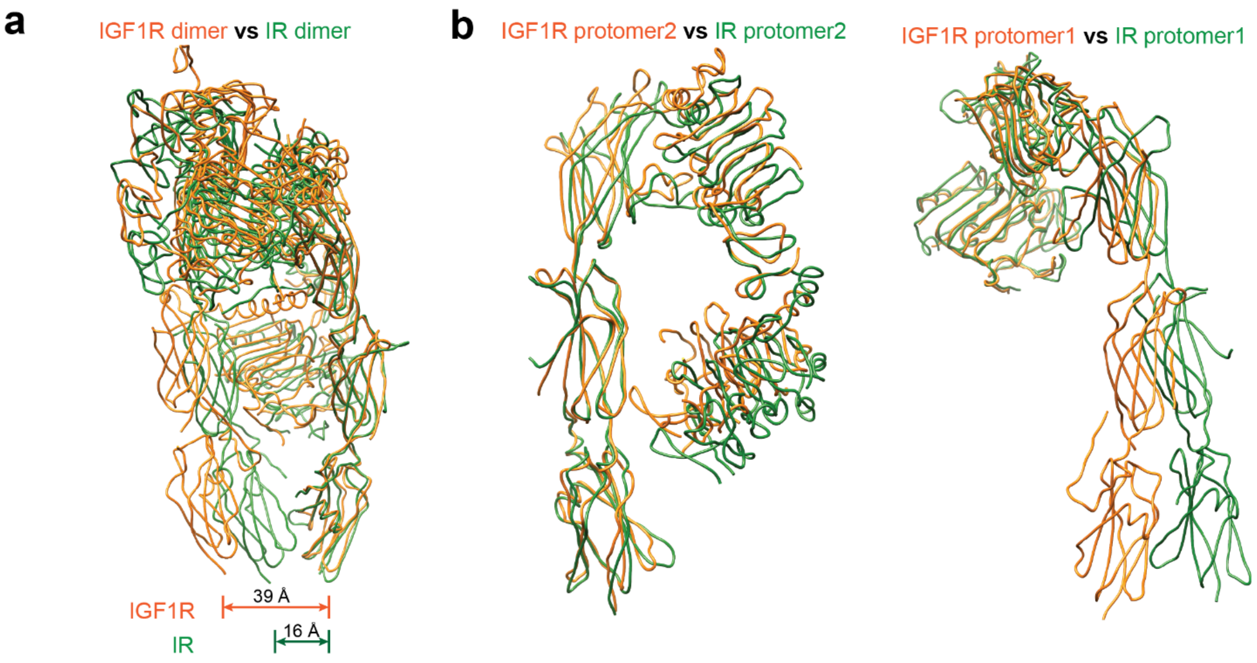

Supplementary Fig. 5 | Structural comparisons between IGF1R and IR. a, Superposition between IGF1R dimer (orange) and IR dimer (green). **b**, Superposition between IGF1R protomers (orange) and IR protomers (green).

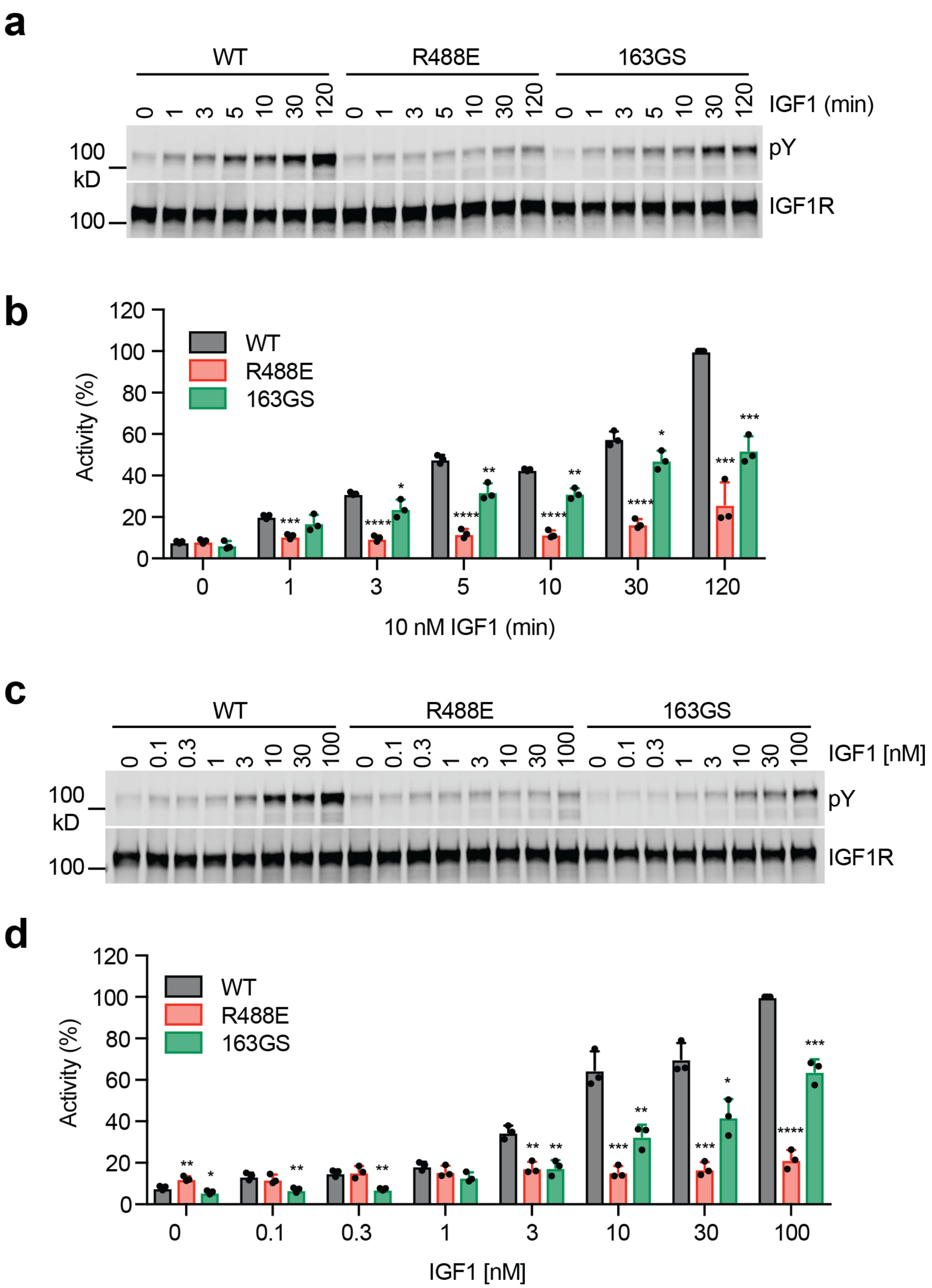

Supplementary Fig. 6 | Time- and dose-dependency of IGF1R activation by IGF1. a, Western blots of time-dependent IGF1R autophosphorylation in 293FT cells expressing Myc-tagged IGF1R WT or mutants. **b**, Quantification of the Western blot data shown in **a** (Mean ±

SD). Each experiment was repeated three times. Significance calculated using two-tailed students *t*-test; *p<0.05, **p<0.01, ***p<0.001, and ****p<0.0001. **c**, Western blots of dose-dependent IGF1R autophosphorylation in 293FT cells expressing Myc-tagged IGF1R WT or mutants. **d**, Quantification of the Western blot data shown in **c** (Mean ± SD). Each experiment was repeated three times. Significance calculated using two-tailed students *t*-test; *p<0.05, **p<0.01, ***p<0.001, and ****p<0.0001. Source data are provided as a Source Data file.

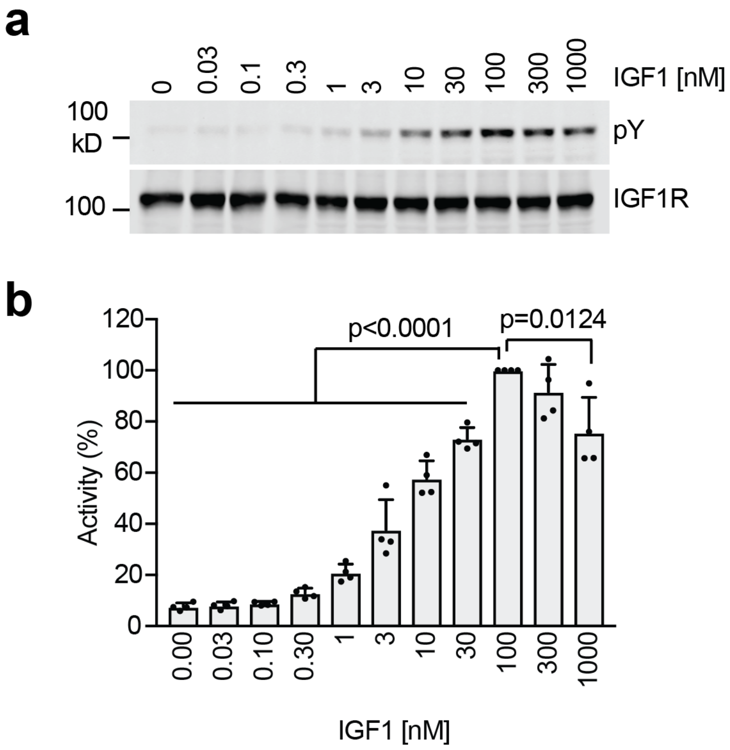

Supplementary Fig. 7 | Dose-dependency of IGF1R activation by IGF1. a, Western blots of IGF1-dependent IGF1R autophosphorylation in 293FT cells expressing Myc-tagged IGF1R WT. **b**, Quantification of the Western blot data shown in **a** (Mean ± SD). Each experiment was repeated four times. Significance calculated using two-tailed students *t*-test. Source data are provided as a Source Data file.

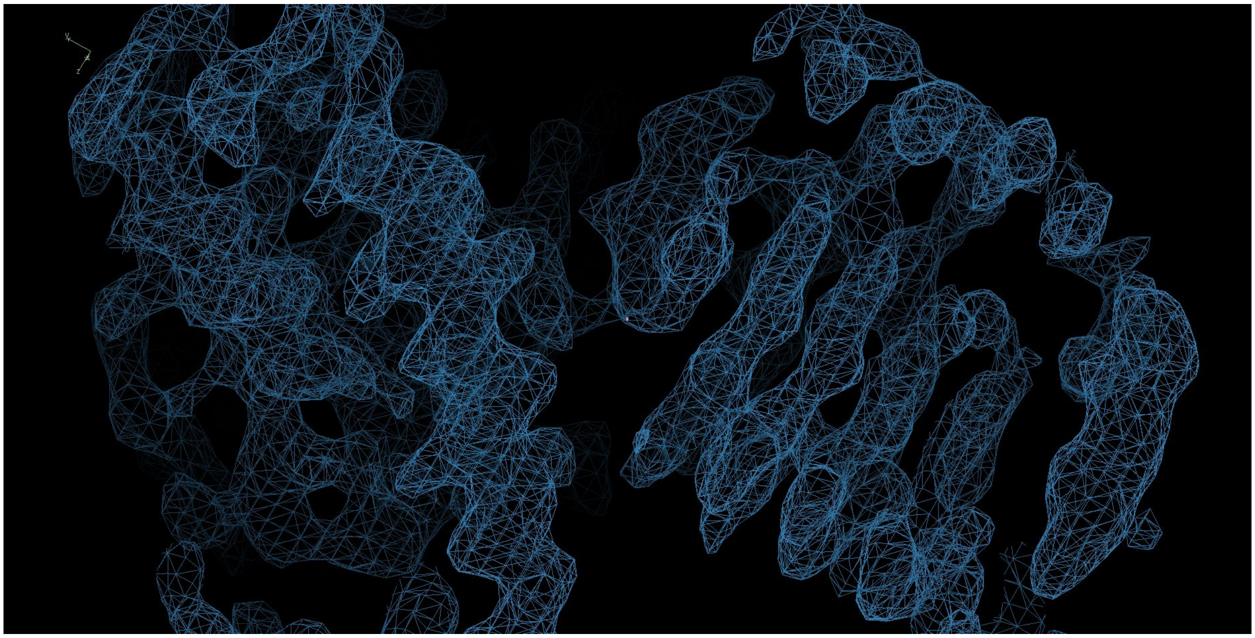

Supplementary Fig. 8 | A portion of the cryo-EM density displayed at a contour level of 0.025.

Supplementary Table 1. List of plasmids.

Plasmid name	NCBI Reference Sequence Name
pCS2-human IGF1R-WT-Myc	NM_000875.5
pCS2-human IGF1R-WT	NM_000875.5
pCS2-human IGF1R-F701A-Myc	NM_000875.5
pCS2-human IGF1R-F701A	NM_000875.5
pCS2-human IGF1R-D1105N-Myc	NM_000875.5
pCS2-human IGF1R-R10A-Myc	NM_000875.5
pCS2-human IGF1R-F90A-Myc	NM_000875.5
pCS2-human IGF1R-Y487A-Myc	NM_000875.5
pCS2-human IGF1R-R488E-Myc	NM_000875.5
pCS2-human IGF1R-N529A-Myc	NM_000875.5
pCS2-human IGF1R-D531A-Myc	NM_000875.5
pCS2-human IGF1R-P673G4-Myc	NM_000875.5
pCS2-human IGF1R-delta 3C-Myc	NM_000875.5
pCS2-human IGF1R-K164A-Myc	NM_000875.5
pCS2-human IGF1R-Y171A-Myc	NM_000875.5
pCS2-human IGF1R-delta 163-174-Myc	NM_000875.5
pCS2-human IGF1R-163GS-Myc	NM_000875.5
pCS2-human IGF1R-T166A-Myc	NM_000875.5
pCS2-human IGF1R-N169A-Myc	NM_000875.5
pEZT-BM-mouse IGF1R-1-1262, Y951A, D1107N	NM_010513.2
pEZT-BM-mouse IGF1R WT	NM_010513.2
