# Supplementary material for: Structural basis of the activation of type 1 insulin-like growth factor receptor
Source: Nat Commun. 2019 Oct 8;10:4567. doi: 10.1038/s41467-019-12564-0 (PMC6783537; doi:10.1038/s41467-019-12564-0)
Supplement: Supplementary file 3 — Reporting Summary [file 41467_2019_12564_MOESM3_ESM.pdf]

nature research

Corresponding author(s): Eunhee Choi, Hongtao Yu, Xiao-chen Bai

Last updated by author(s): Sep 5, 2019

Reporting Summary

Nature Research wishes to improve the reproducibility of the work that we publish. This form provides structure for consistency and transparency in reporting. For further information on Nature Research policies, see Authors & Referees and the Editorial Policy Checklist.

Statistics

For all statistical analyses, confirm that the following items are present in the figure legend, table legend, main text, or Methods section.

n/a	Confirmed	
☐	☒	The exact sample size (*n*) for each experimental group/condition, given as a discrete number and unit of measurement
☐	☒	A statement on whether measurements were taken from distinct samples or whether the same sample was measured repeatedly
☐	☒	The statistical test(s) used AND whether they are one- or two-sided<br>*Only common tests should be described solely by name; describe more complex techniques in the Methods section.*
☒	☐	A description of all covariates tested
☒	☐	A description of any assumptions or corrections, such as tests of normality and adjustment for multiple comparisons
☐	☒	A full description of the statistical parameters including central tendency (e.g. means) or other basic estimates (e.g. regression coefficient) AND variation (e.g. standard deviation) or associated estimates of uncertainty (e.g. confidence intervals)
☐	☒	For null hypothesis testing, the test statistic (e.g. *F*, *t*, *r*) with confidence intervals, effect sizes, degrees of freedom and *P* value noted<br>*Give P values as exact values whenever suitable.*
☒	☐	For Bayesian analysis, information on the choice of priors and Markov chain Monte Carlo settings
☒	☐	For hierarchical and complex designs, identification of the appropriate level for tests and full reporting of outcomes
☒	☐	Estimates of effect sizes (e.g. Cohen's *d*, Pearson's *r*), indicating how they were calculated

Our web collection on statistics for biologists contains articles on many of the points above.

Software and code

Policy information about availability of computer code

Data collection	EPU 2.1 (Thermo Scientific)
Data analysis	RELION3, MotionCorr2.1, GCTF1.06, pClamp 9, Coot0.8.8, ResMap1.1.4, PHENIX1.11.1, Chimera1.11.2, All software used is commercially or publicly available and described in the Methods section. Data displayed in graphs were analyzed using GraphPad Prism 7.0 d (GraphPadSoftware Inc.)

For manuscripts utilizing custom algorithms or software that are central to the research but not yet described in published literature, software must be made available to editors/reviewers. We strongly encourage code deposition in a community repository (e.g. GitHub). See the Nature Research guidelines for submitting code & software for further information.

Data

Policy information about availability of data

All manuscripts must include a data availability statement. This statement should provide the following information, where applicable:

- Accession codes, unique identifiers, or web links for publicly available datasets
- A list of figures that have associated raw data
- A description of any restrictions on data availability

The datasets generated during and/or analysed during the current study are available in the PDB and EMDB repository. No restrictions on data availability.

Field-specific reporting

Please select the one below that is the best fit for your research. If you are not sure, read the appropriate sections before making your selection.

☒ Life sciences ☐ Behavioural & social sciences ☐ Ecological, evolutionary & environmental sciences

For a reference copy of the document with all sections, see nature.com/documents/nr-reporting-summary-flat.pdf

Life sciences study design

All studies must disclose on these points even when the disclosure is negative.

Sample size	Sample sizes are given in the manuscript. All binding and cellular assays were repeated at least three times.
Data exclusions	Only the particles that showed homogeneous conformation were selected in the final reconstruction.
Replication	All experimental findings were reliably reproduced.
Randomization	For resolution estimation, all particles were randomly split into two groups.
Blinding	The investigators were blinded to group allocation during data collection and analysis.

Reporting for specific materials, systems and methods

We require information from authors about some types of materials, experimental systems and methods used in many studies. Here, indicate whether each material, system or method listed is relevant to your study. If you are not sure if a list item applies to your research, read the appropriate section before selecting a response.

Materials & experimental systems

n/a	Involved in the study	
☐	☒	Antibodies
☐	☒	Eukaryotic cell lines
☒	☐	Palaeontology
☒	☐	Animals and other organisms
☒	☐	Human research participants
☒	☐	Clinical data

Methods

n/a	Involved in the study	
☒	☐	ChIP-seq
☒	☐	Flow cytometry
☒	☐	MRI-based neuroimaging

Antibodies

Antibodies used	Anti-IGF1R-pY1135/1136 (19H7, Cell signaling; labeled as pY)<br>Anti-MYC (9E10, Roche; labeled as IGF1R)<br>Anti-rabbit immunoglobulin G (IgG) (H+L) (Dylight 800 conjugates)<br>Anti-mouse IgG (H+L) (Dylight 680 conjugates) (Cell signaling)<br>Anti-mouse IgG (H+L) (Dylight 800conjugates) (Cell signaling)
Validation	We used IR-pY1135/1136 (19H7, Cell signaling) and Myc (9E10, Roche) antibodies. We detected a band with expected molecular weight when these antibodies were used to probe human 293FT cell lysates.

Eukaryotic cell lines

Policy information about cell lines

Cell line source(s)	293FT (R70007) cell line was purchased from Invitrogen.
Authentication	We freshly purchased 293FT (R70007) cell lines from Invitrogen and did not perform the additional authentication process.
Mycoplasma contamination	Negative for mycoplasma
Commonly misidentified lines<br>(See ICLAC register)	No commonly misidentified cell lines were used.
